# Coastal Phytoplankton Response to Acidification and Warming Under Differing Levels of Nutrient Availability

**DOI:** 10.3390/microorganisms14050989

**Published:** 2026-04-28

**Authors:** Cliff S. Law, Neill Barr, Kim Currie, Stacy Deppeler, Peter W. Dillingham, Mark P. Gall, Andrew Marriner, Kiri McComb, Judith Murdoch, Lisa Northcote, Karl Safi

**Affiliations:** 1 Earth Sciences New Zealand (Formerly National Institute for Water and Atmospheric Research), Wellington 6023, New Zealand; neill.barr@earthsciences.nz (N.B.); s.deppeler@earthsciences.nz (S.D.);; 2 Department of Marine Science, University of Otago, Dunedin 9016, New Zealand; judith.murdoch@otago.ac.nz; 3 Earth Sciences New Zealand, Dunedin 9016, New Zealand; 4 Department of Mathematics and Statistics, University of Otago, Dunedin 9016, New Zealand; peter.dillingham@otago.ac.nz; 5 Isotrace New Zealand Ltd., Dunedin 9016, New Zealand; kmccomb@oritain.com; 6 Earth Sciences New Zealand, Hamilton 3216, New Zealand; karl.safi@earthsciences.nz

**Keywords:** phytoplankton, ocean acidification, warming, nutrients, mesocosm experiment, diatom, small flagellates, time series, coastal

## Abstract

Ocean acidification and warming will alter phytoplankton biomass and composition, yet despite numerous studies, there are few consistent responses on which to base predictions. To determine the responses of chlorophyll-*a* and phytoplankton size and composition to predicted lower pH (−0.33 to −0.5) alone, and also combined with elevated temperature (+2.5–3.5 °C), two mesocosm experiments were carried out in austral spring and autumn in temperate New Zealand coastal waters. Lower pH alone had no effect on chlorophyll-*a* in either experiment and, as the treatment pH was lower than the pH minimum recorded in a parallel four-year time series, this lack of response in chlorophyll-*a* was not attributable to prior in situ exposure. Conversely, chlorophyll-*a* increased under lower pH and warming in both experiments, with the large (>20 µm) phytoplankton size fraction showing opposing responses under nutrient deplete and replete conditions. Diatom biomass also increased in both treatments when nutrient availability was maintained, with a dominant pennate species *Cylindrotheca clostridium* emerging. The results highlight the value of contextual time series for experimental interpretation, and also the importance of assessing warming and acidification together using regionally representative nutrient concentrations, for prediction of coastal phytoplankton response to climate change.

## 1. Introduction

The increasing atmospheric CO_2_ burden has motivated a number of experimental studies over the last two decades examining the responses of phytoplankton to changes in pH and the carbonate system associated with ocean acidification and related increases in temperature [1]. As photosynthesis by phytoplankton is reliant upon dissolved CO_2_ (pCO_2_), ocean acidification may impact phytoplankton biomass, productivity and community structure; yet, to date, experimental findings are inconsistent, with responses to elevated pCO_2_ ranging from minor effects through to major community reorganisation [2,3,4]. This variability arises in part from the underlying physiological mechanism by which different phytoplankton species obtain carbon. CO_2_ supply to the cell is dependent on diffusion and so is influenced by cell size, with smaller phytoplankton less dependent on pCO_2_ due to their larger surface area:volume ratio. Conversely, larger cells may be diffusion-limited and often use Carbon Concentrating Mechanisms (CCMs) to maintain supply [5]. Diatoms are particularly reliant upon CCMs and may benefit from energy saved from downregulation of this mechanism under elevated pCO_2_ [6,7]; however, this benefit may be offset by the additional metabolic cost of maintaining intracellular pH [8], with the balance between stimulatory and inhibitory physiological responses resulting in a “universal reaction norm” for [H^+^] [9]. As this norm varies with taxa and groups, analogous to temperature norms [10], this may account for the range of responses, in both sign and magnitude, reported in ocean acidification studies [4]. However, variation in response to elevated pCO_2_ may also result from initial differences in biomass, bloom state, and community composition [11] due to variation in environmental drivers such as nutrient availability, light and grazing that reflect spatio-temporal variation across seasons and regions. In addition, differing experimental frameworks, from unispecific culture experiments that focus on physiological and mechanistic responses to large volume mesocosms that simulate natural food web interactions and environmental conditions, have differing levels of complexity that may influence outcome.

Temperature is a primary control of metabolic processes [12], and warming may manifest via phytoplankton physiological processes as changes in productivity, cell composition and size, phenology and diversity [13]. The indirect effects of warming may be equally influential by altering light environment, nutrient availability, interspecific competition and top–down control by grazers, with resulting impacts on phytoplankton biomass and community composition [1]. Yet, despite a variety of direct and indirect impact pathways, warming has received arguably less attention than ocean acidification and so, despite many valuable insights, the focus on ocean acidification in many experiments may limit prediction of future ecosystems. Acidification will occur in parallel with warming, and the common source and interaction of these two climate drivers requires their combined effect to be established [14]. For example, this interaction may have variable effects on phytoplankton processes that cannot be anticipated from studies of the individual drivers in isolation, with synergistic responses often dominating when the two climate drivers are combined [15,16]. To date phytoplankton sensitivity to multiple stressors has been primarily tested in cultures and small-volume systems [17], with few mesocosm studies of community-level response, in part due to the challenge of replication with multiple drivers [18]. As a result, the integrated influence of these two drivers on trophic interactions in a natural community are often precluded, even though this may ultimately determine net ecosystem response in the future ocean.

Acidification and warming experiments have generally focused on coastal waters partly for logistical reasons but also because local and regional anthropogenic and terrestrial influences drive pCO_2_ variations and extremes that exceed that of the open ocean [19]. Indeed, coastal pH variability may exceed the projected perturbation associated with climate change over short spatial and temporal scales. Furthermore, coastal waters experience variation in nutrient availability, as a result of terrestrial input, heterotrophy and sediment–water exchange which, when combined with elevated insolation and temperature variation in shallow waters, places coastal regions at the nexus of warming, eutrophication and ocean acidification [20,21,22]. Consequently, determining response to future change in climate-related drivers necessitates experimentation and interpretation against a well-characterised understanding of the natural variability of the coastal system [23]. For example, there is evidence that phytoplankton community response to acidification is sensitive to nutrient state [24,25,26], and so experimental conditions need to reflect in situ nutrient availability and variability. Similarly, experimental perturbation of pH or temperature should be based upon and interpreted with respect to regional variation for meaningful future prediction.

The following study takes these factors into consideration in two mesocosm experiments under differing bloom states in different seasons to examine how phytoplankton communities in temperate New Zealand coastal waters may respond to projected future acidification and warming. The results are interpreted with respect to in situ variability in pH and temperature by reference to a parallel four-year time series at the same location. The responses in chlorophyll-*a* concentration and phytoplankton size and diversity are also assessed in relation to nutrient availability, with one experiment carried out under nutrient-deplete conditions and the other with nutrients maintained at ambient concentrations. A further objective of the study was to discriminate between the response to lower pH alone, and elevated temperature and lower pH in combination, to determine their interactive effects for prediction of future phytoplankton state.

## 2. Materials and Methods

### 2.1. Mesocosm Framework

**Figure 1:**
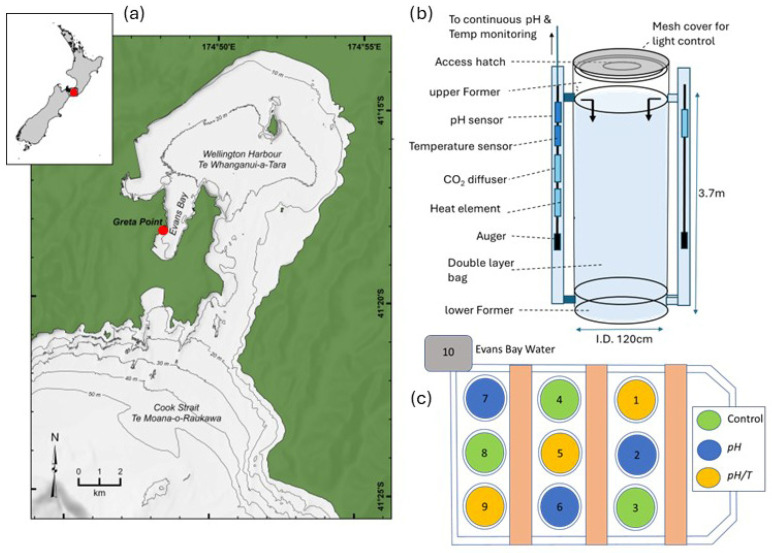
(**a**) Location of mesocosm experiments and pH time series at Greta Point in Ākautangi Evans Bay (red dot), in relation to Wellington Harbour and New Zealand (inset); (**b**) mesocosm bag construction, with water flow direction indicated by arrows; (**c**) experimental layout of nine mesocosm bags in the pond, with treatments and controls indicated by colour, and ambient Ākautangi Evans Bay water supply indicated as 10.

Two mesocosm experiments were carried out in austral autumn 2016 and spring 2018, using coastal water from Ākautangi Evans Bay, a semi-enclosed bay at the southern end of Wellington Harbour, New Zealand (Figure 1a). Other aspects of phytoplankton response in these mesocosm experiments are reported elsewhere [27,28,29].

Both experiments incorporated nine 3.7 m long mesocosm bags, each filled with coastal seawater from a depth of ~15 m, with the containment pond (Figure 1c) continually flushed with bay water to maintain ambient temperature. The nine mesocosm bags were composed of a prefabricated cylindrical ‘bag’ (1.2 m diameter by 3.5 m height; water volume 4.2 m^3^), made of reinforced polyethylene ‘light blocking’ canvas, with upper and lower polyethylene (6 mm white) cylindrical formers (1.2 m diameter and 0.5 m height) attached to either end of the open canvas ‘bag’ (Figure 1b). The interior surface of the bags was white to reflect and diffuse downwelling light within the mesocosm. The upper and lower formers were connected by two 80 mm diameter PVC side-pipes (3.5 m in length) to maintain a semi-rigid cylindrical form once deployed, and also to facilitate turnover between bottom and surface waters. The lower former had a closed base with ballast attached externally while the upper former had a 1.2 m diameter × 6 mm thick clear Perspex lid enclosing a 0.34 m^3^ air headspace (above the water line) that allowed light penetration and access for water sampling via a hatch. The bags were also suspended from a scaffold frame to maintain their shape and volume. The bags were also enclosed by a layer of 3 mm underfloor heating insulation to facilitate temperature control in the warmer treatments, which also excluded diffused-light penetration through the bag walls.

### 2.2. Experimental Design

The bags were initially filled simultaneously and overflowed for a minimum of 24 h to ensure thorough flushing and uniform conditions. The target pH and temperature in the treatments (Table 1) were based on future projections for the New Zealand region for the year 2100 using an Earth System Model with the RCP8.5 emission scenario [30]. pH and temperature in the treatments were slowly adjusted over 24–36 h from initial values to the target pH and temperature. A consistent framework was used in both experiments with comparison of three control replicates with three replicates of one treatment in which only pH was adjusted (identified hereon as *pH*), and a second treatment with three replicates in which both pH and temperature were adjusted (hereon identified as *pH*/*T*). One of the triplicate bags for the control and two treatments were placed in each of the three rows in the pond to accommodate any variation in ambient light (Figure 1c). As all replicate bags were co-located, inference is limited to treatment effects under the environmental conditions at the time of sampling at the site, and results may differ if the experiment was repeated under different environmental conditions. Although the experimental design was not full factorial, it distinguished the individual influence of pH, whilst also enabling assessment of the response to combined warming and lower pH which was representative of future conditions.

Seawater temperature and pH in each mesocosm were controlled by a 3 kW heater element and a CO_2_ permeable diffuser coil, respectively, which were located within each side-pipe. Water circulation and mixing were achieved by a pump in ND and an auger in NR, both situated in the side-pipe, which ensured vertical uniformity and prevented stratification in the bags. Temperature and pH were monitored continuously by plastic-coated thermistor temperature and Sensorex (Garden Grove, CA, USA) S150 pH probes, respectively, located upstream of the controlling components in the vertical side-pipes. Both parameters were continuously adjusted and maintained in the treatments using a Labview-based control system, with changes of 0.1 °C and 0.01 pH units detected by the pH and temperature control system, which activated automatic feedback adjustment to achieve target values for temperature and pH (Figure 1). The effect of differing nutrient availability was tested, with the first experiment in austral autumn 2016, ND (Nutrient Deplete), receiving no nutrient addition, whereas nutrients were added on a daily basis during the second experiment in spring 2018 to replicate in situ nutrient availability in NR (Nutrient Replete). The nutrient additions in NR (Table 1) were administered at 16:00–17:00 each day as a single spike into the side-pipe outlet flow at the surface to ensure rapid mixing and uniformity.

Monitoring of selected parameters was achieved using an in-line system that pumped water from each bag once every hour through an Exosonde, incorporating sensors for temperature, pH, dissolved oxygen, salinity, and fluorescence. Measurements of these variables at 90 min intervals in each bag enabled issues, such as power or sensor failure, to be rapidly identified. Discrete water samples were obtained routinely between 09:00 and 10:00 each day by removal of 25 L of seawater by gravity feed; this volume, which was less than 1% of total bag volume, was replaced with ambient bay water at the same time for all bags, which introduced some uncontrolled variation. Core parameters, including nutrients and chlorophyll-*a* concentration (Chl-*a*), were sampled each day during the 18-day ND experiment, and every second day during the 20-day NR experiment, with all other parameters sampled at 2–4-day intervals.

### 2.3. Ancillary Parameters

#### 2.3.1. Nutrients

A total of 500 mL of water was sampled on a daily basis and filtered through 25 mm GF/F filters with the bottles sealed with parafilm and stored at −20 °C. Nutrient samples were measured in units of µmol/L on a SEAL AA3 autoanalyser, a 4-channel nutrient system consisting of an XY2 sampler, two pumps, two chemistry modules, two colorimeters and a JASCO Fluorimeter. Nitrate + nitrite was measured using colorimetric reagents—sulfanilamide and N-(1-Naphthyl) ethylenediamine dihydrochloride (NEDDE) in combination with a cadmium reduction column and SEAL Method G-172, with the resultant complex measured at 550 nm or 520 nm. Dissolved reactive phosphorus was measured using colorimetric reagents—ammonium molybdate, antimony potassium tartrate and ascorbic acid and SEAL Method G-297, with the resulting complex measured at 880 nm. Silica is measured using colorimetric reagents—ammonium molybdate, oxalic acid and ascorbic acid and SEAL Method G-177, with the generated coloured complex measured at 820 nm. Ammonia was measured using Fluorometric reagents—o-phthalaldehyde (OPA), sodium sulfite, and disodium tetraborate buffer and SEAL Method G-327-05 Rev 8, with fluorescence measured at 460 nm following excitation at 370 nm.

#### 2.3.2. Carbonate System

To establish the inter-annual variation in pH and temperature, water samples of 1 L volume were routinely collected at the Evans Bay intake and poisoned using mercuric chloride, with a total of 133 water samples obtained at bimonthly intervals between January 2015 and July 2019, as part of the 4-year time series data [31]. During the two mesocosm experiments, 1 L water samples were also collected every second day from each bag to characterise the carbonate system and used to determine pH via measurement of Dissolved Inorganic Carbon (DIC) and Total Alkalinity (TA). DIC was measured using coulometric analysis of the carbon dioxide evolved from the acidified sample as described in [32]. Briefly, an accurately known volume of the seawater sample is dispensed into a stripper containing phosphoric acid. The carbonate ions, bicarbonate ions and carbonic acid in the seawater react with the acid to form carbon dioxide which is then purged with carrier gas and transferred into a reaction cell where the carbon dioxide is determined by coulometry. Certified Reference Material (A Dickson, Scripps Institute of Oceanography) was analysed with each batch of samples, with accuracy and precision of ±1 mmol/kg. TA was measured using a closed cell potentiometric titration [32], with an accurately known volume of seawater introduced into a reaction cell which was filled and sealed, with the initial emf (pH) of the sample determined. The sample was then titrated with hydrochloric acid, with the volume of HCl added and emf (pH) measured at each titration point. The resulting titration curve was then analysed by comparison with a theoretical curve using a least squares minimisation method. Certified Reference Material (A Dickson, Scripps Institute of Oceanography) was analysed with each batch of samples, with an accuracy and precision of ±2 mmol/kg.

#### 2.3.3. Phytoplankton

Total chlorophyll-*a* concentration (Chl-*a*, mg/m^3^) was determined every day during ND, and every second day during NR, by filtering a 250 mL sample through a GF/F filter, which was then folded, snap-frozen in Liquid Nitrogen, and stored at −80 °C prior to extraction in 90% acetone and analysis using a Turner Design fluorometer [33]. The Chl-*a* concentration of different size fractions (0.2–2 µm, 2–5 µm, 5–20 µm, >20 µm) was determined every second day by filtering 500 mL through polycarbonate filters of sequentially decreasing pore size to obtain information on changes in the size spectrum of the phytoplankton.

Phytoplankton community composition and abundance of cells > 5 µm were determined using optical microscopy. One litre of water was preserved every 2–4 days during the experiments, using Lugol’s solution, and subsequently examined using optical microscopy, as described in [34], and references therein. Subsamples of 50 to 150 mL were settled in measuring cylinders, with settling times of 0.5 h/millimetre of settling column depth, and the supernatant was then syphoned off, with the remaining 10 mL transferred to Utermohl chambers and resettled. Samples were then enumerated using a Leica DMI3000B inverted microscope at 100× to 600× magnification [35]. Where possible, all abundant organisms were identified to genus or species level before being counted. Biovolume was calculated for each species, using formulae representing the geometrical solids that approximated cell shape and adjusted for cell shrinkage following [36], with some modifications following [37,38]. Phytoplankton carbon (mg C/m^3^) was then calculated using the conversion equations of [39] for diatoms and dinoflagellates, and [40] for other low-biomass groups including small flagellates (<~5 µm), Raphidophyceae, Prymnesiophyceae, Cryptophyceae, Chrysophyceae, Euglenoids, and Monads. *Cylindrotheca closterium* was identified following standard morphological criteria for the genus *Cylindrotheca*.

Samples for Total Particulate Carbon and nitrogen were collected every second day during ND and every fourth day during NR by filtering 500 mL of sample water through a pre-combusted 25 mm GF/F filter with storage at −20 °C. Analysis was carried out on a DELTA V Plus continuous flow isotope ratio mass spectrometer linked to a Flash 2000 elemental analyser (EA-IRMS) via a ConFlo IV, and using a MAS 200 R autosampler (Thermo-Fisher Scientific, Bremen, Germany) at the NIWA Environmental and Ecological Stable Isotope Facility in Wellington, New Zealand. Carbon and nitrogen content were calculated from thermal conductivity detector values during EA-IRMS analysis.

#### 2.3.4. Data Analysis

GAMMs (Generalised Additive Mixed Models) were used to identify temporal variations in Chl-*a*, size-fractionated Chl-*a* and phytoplankton groups in the treatments relative to the control over the duration of each experiment, as in [27]. Datasets were provisionally tested for normality using a Normal Q-Q plot to ensure that each dataset was normally distributed. Three models were then generated with each accounting for repeated measures with inclusion of an auto-correlation term for each treatment replicate, as in [41].

- Model 1 (Null model) provided a global smooth across the experiment with no consideration of different treatments using predictors of Day Number and the auto-correlation term;
- Model 2 provided independent smooths across each experiment for each treatment, with predictors of Day Number subset by Treatment and the auto-correlation term, so representing individual processes over time in each treatment;
- Model 3 provided both global and independent smooths across days for each treatment using predictors of Day Number, Day Number subset by Treatment ID, and the auto-correlation term, representing one underlying process affected by individual treatment effects.

The relative information loss between models was assessed using the Akaike Information Criteria (AIC) with method “ML” for model comparison. When models 2 or 3 were chosen, additional assessment was carried out by determining smoothing functions that best represented the difference between each treatment and the control by testing the deviation from zero [42]. If the AIC indicated a treatment effect, the deviation from zero was assessed using a Wald’s test.

The GAMMs approach facilitated comparison of temporal trends across each experiment but was less suitable for identifying treatment effects that became increasingly dominant with time, and also for comparing means within different phases of the experiments. Consequently, differences between the control and treatments were also assessed by comparison of mean values for Phases 2 and 3 (see phase definition in Section 3.2.2 below). This was carried out using *t*-test and Wilcoxon rank sum approaches, with the latter applied for non-normal behaviour in certain cases and adjusted for family-wise error using the Holm–Bonferroni correction to reduce the number of Type I errors (false positives). As these mean estimates incorporated temporal variation of three replicates over a 6–8-day period within each phase in addition to natural population variance, *p <* 0.1 was utilised as suggested evidence of differences.

## 3. Results

### 3.1. pH Time Series

**Figure 2:**
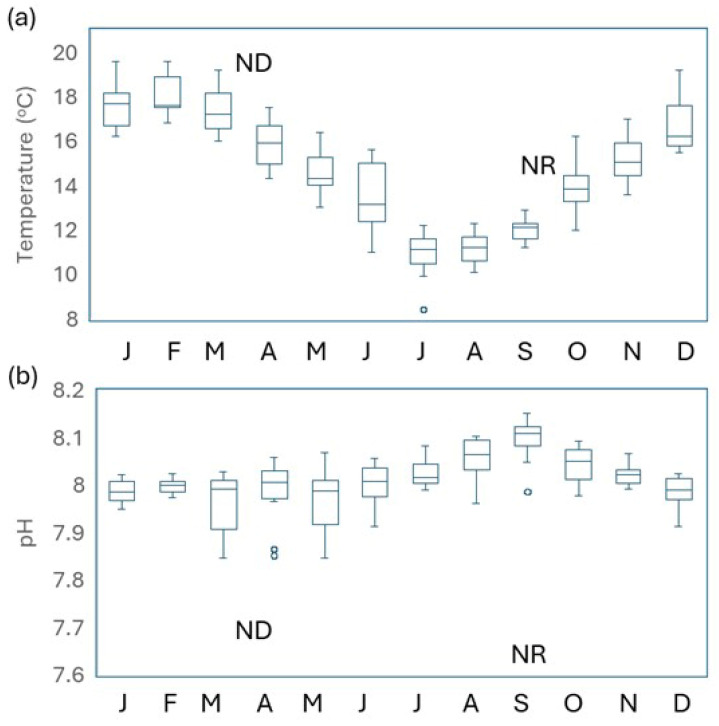
Box and Whisker plots of (**a**) temperature and (**b**) pH for the 4-year time series in Ākautangi Evans Bay, which encompassed the two experiments. The horizontal blue line in each month indicates the mean, the blue box the quartile values, and outliers shown as small circles. The labels (ND, NR) indicate the treatment temperature (in *pH*/*T)* and pH (in *pH* and *pH*/*T)* in the respective experiment.

Variation in pH and temperature in Evans Bay showed expected seasonal trends in temperature over the four-year time series, ranging from 8.5 °C in the winter to 19.6 °C in the summer (Figure 2 and Figure S1) and a pH maximum of 8.12 in austral spring (September) and minimum of 7.99 in summer–autumn. Variability was largest in early winter for both parameters. Comparison of treatment pH and temperature (Table 1) with the time series data in Figure 2 indicates that the experimental temperature perturbations were within the annual and respective seasonal range, whereas treatment pH was lower than both the seasonal and annual range in both experiments.

**Figure 3:**
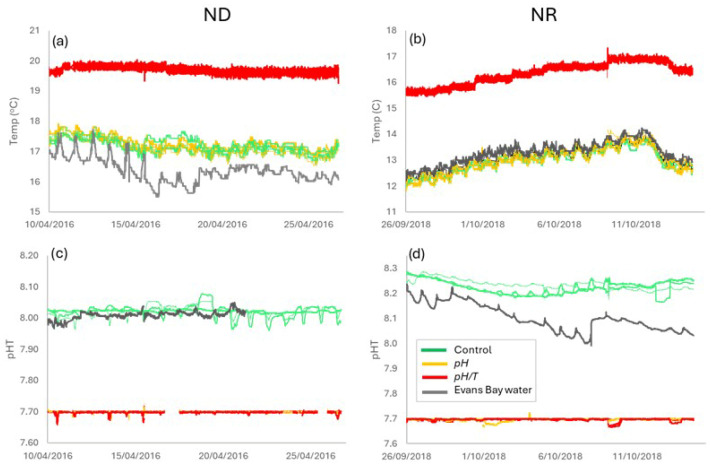
(**a**,**b**) Water temperature (°C), and (**c**,**d**) pH in the three replicates of the control (green), *pH* (orange) and *pH*/*T* (red) treatments, and in Ākautangi Evans Bay water (black), during ND (**left**) and NR (**right**) against time (date). Bay water pH is not shown from Day 10 onward in ND due to biofouling of the intake. Note the difference in pH and temperature range on the vertical axis between the two experiments.

Continuous measurement throughout the two experiments confirmed that the temperature of the control and *pH* treatment tracked ambient temperature fluctuations in Ākautangi Evans Bay, with corresponding diurnal oscillation (Figure 3), whereas maintenance of the target temperature in *pH*/*T* precluded this fluctuation. Similarly, maintenance of pH in the two treatments eliminated variability, whereas pH in ambient Bay water and the controls exhibited diurnal oscillation. Although diel fluctuations in pH and temperature are natural features of coastal waters and may moderate phytoplankton responses relative to uniform conditions [43], the diurnal pH variability in the control did not exceed 0.06 and so was minor relative to the 0.3–0.5 pH decrease in the treatments. Similarly, the diurnal variation in the bay water temperature was <1 °C relative to the variation of ~0.25 °C in *pH*/*T*. Consequently, the experimental framework maintained pH and temperature at constant levels representative of the future ocean for the duration of the experiment, and so provided a hybrid framework between small-scale laboratory studies with fixed temperature and pH [16] and in situ mesocosm studies in which pH is adjusted initially or intermittently [24].

### 3.2. Experiment Response

#### 3.2.1. Nutrients

**Figure 4:**
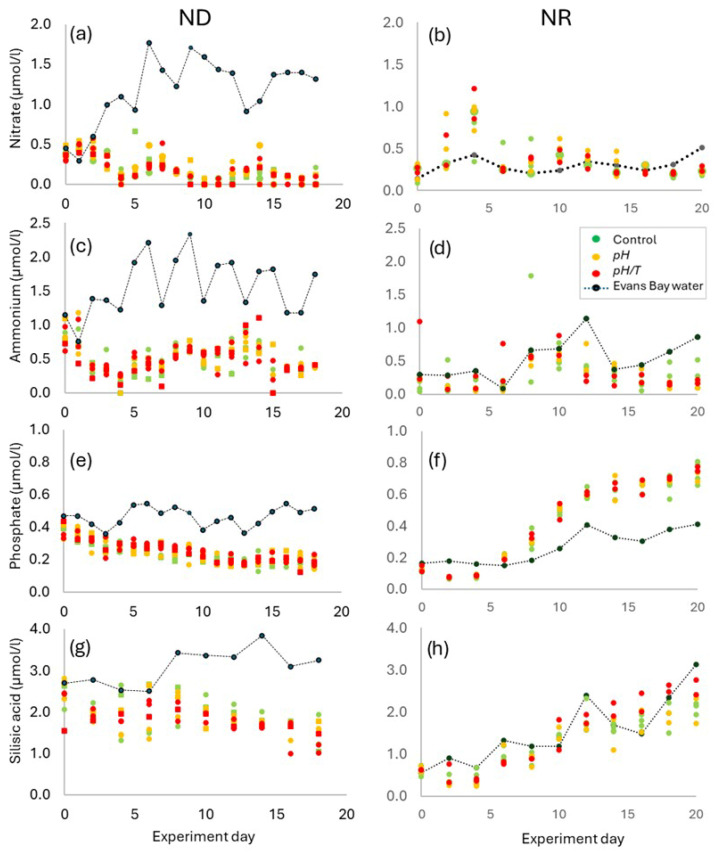
Nutrient concentrations (µmol/L) for (**a**,**b**) nitrate, (**c**,**d**) ammonium, (**e**,**f**) phosphate, (**g**,**h**) silicic acid, in the three replicates for the control (green symbols), *pH* (orange symbols) and *pH*/*T* (red symbols) treatments, and in Ākautangi Evans Bay water (black symbols and line), during ND (**left**) and NR (**right**) against experiment day.

The initial nutrient status of the two experiments differed, as ND was carried out during austral autumn and NR in austral spring (Table 1, Figure 4). In ND initial concentrations of nitrate were 0.6–1.0 µmol/L and phosphate 0.35–0.44 µmol/L, with silicic acid (2–2.7 µmol/L) and ammonia (0.6–1.1 µmol/L) showing variability between replicates. Both nitrogen species were drawn down to <0.3 µmol/L by Day 4 but rallied to 0.5 µmol/L by Day 6 with ammonium increasing to Day 14 whilst nitrate declined to undetectable levels by Day 9–10. Both silicic acid and phosphate declined at a uniform rate throughout ND with concentrations of 1.2 and ~0.2 µmol/L, respectively, by the end of the experiment. There were no differences in nutrient concentrations between the controls and treatments in ND. Ambient bay water concentrations fluctuated throughout ND but were generally higher for all nutrients relative to the controls and treatments, and hence ND represented a “nutrient-deplete” scenario.

Initial nitrate and phosphate concentrations were similar in NR, but daily nutrient additions elevated nitrate concentration to ~1.0 µmol/L on Day 4, after which nitrate stabilised at 0.2 µmol/L (Figure 4). Conversely, the daily additions resulted in a steady increase in phosphate from 0.1 to 0.7 µmol/L by the end of the experiment. Silicic acid was initially lower than in ND at 0.6 µmol/L, as a result of the diatom bloom when NR started, but daily additions resulted in a steady increase to 2 µmol/L. Ammonium again showed greater variability with initial concentrations of <0.2 µmol/L increasing to 0.4–0.8 µmol/L by mid-experiment before declining to 0.1–0.5 µmol/L by the end of NR. As with ND there was no difference in nutrient concentrations between the control and treatments; however, unlike ND, nitrate, ammonium and silicic acid concentrations during NR tracked ambient bay water concentrations from Day 4 onwards, and consequently NR represented a “nutrient-replete” scenario.

#### 3.2.2. Total Chlorophyll

**Figure 5:**
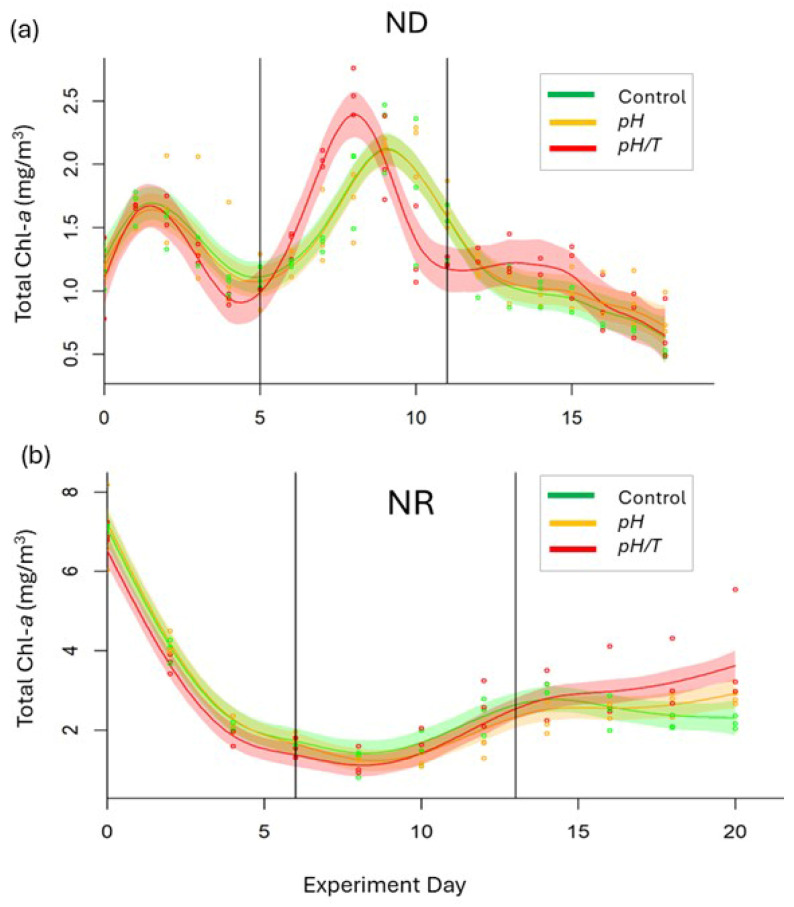
Total Chl-*a* (mg/m^3^) for (**a**) ND and (**b**) NR against experiment day, showing the individual data points (filled circles) from each bag overlain by the GAMM fits (mean and 95% Confidence Interval) for the control (green) and treatments (*pH*, orange, and *pH*/*T*, red). The three phases of each experiment are delineated by the vertical lines. Note that the Chl-*a* concentration scale on the *y*-axis differs between experiments.

Chl-*a* concentration was used to divide both experiments into three phases, with an initial phase (Day 0 to Day 5–6), a mid-phase (Days 6–12), and a final phase (Day 11 onwards). Assessing the results in Phases, rather than at specific timepoints or days, accommodated temporal variation between replicates and different sampling frequency between parameters within each experiment. Phase 1 results were not assessed as responses during this period were driven by initial adjustment of the phytoplankton community to the mesocosm environment from a common initial condition. The two experiments were characterised by different initial bloom states with low Chl-*a* in ND (~1 mg/m^3^) and elevated Chl-*a* (~6.5 mg/m^3^) in NR (Table 1, Figure 5b). Temporal trends in Chl-*a* also differed between experiments, with a Chl-*a* maximum on Days 6–8 in ND (Figure 5a), whereas NR showed a decline in Chl-*a* following the initial bloom, followed by an increase from Day 12 onwards. Common trends were observed between the control and *pH* in both experiments with no differences in Chl-*a,* except for deviation in the final 2–3 days of NR (Figure 5b). Conversely, there were differences in Chl-*a* between the control and *pH*/*T* in ND (Figure 5a), with an earlier (1–2 days, *p* < 0.001, Table S1) and larger Chl-*a* maximum in *pH*/*T* during Phase 2 and a higher Phase 3 mean. There was also a difference between *pH*/*T* and the control (*p* < 0.001) in NR (Figure 5b)*,* with higher Chl-*a* in *pH*/*T* in Phase 3 (3.31 vs control 2.46 mg/m^3^, *p* = 0.01).

#### 3.2.3. Size-Fractionated Chlorophyll

**Figure 6:**
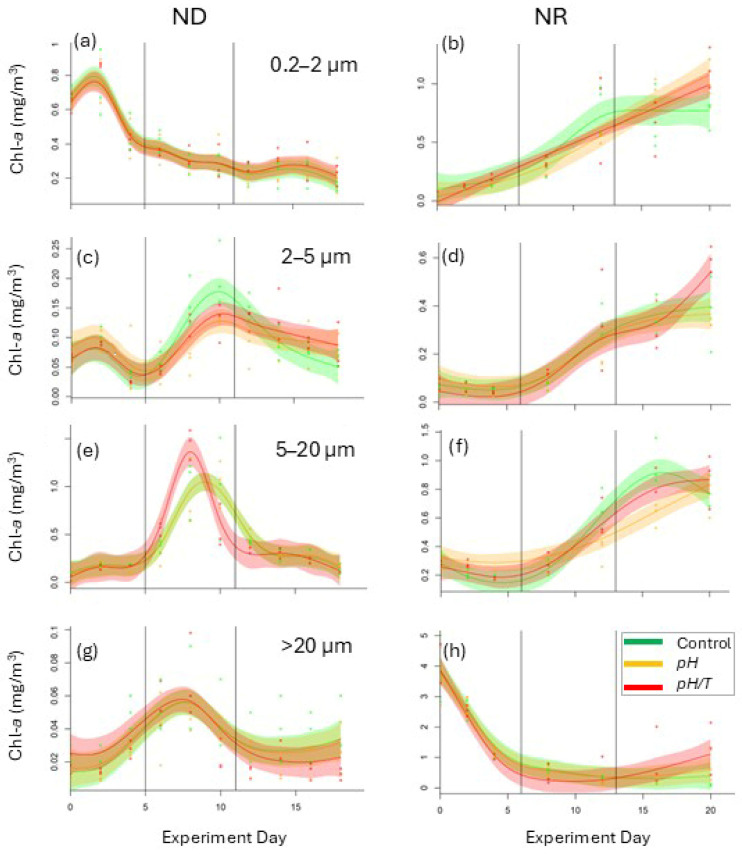
Chl-*a* size fraction (mg/m^3^) response for (**a**,**b**) 0.2–2 µm, (**c**,**d**) 2–5 µm, (**e**,**f**) 5–20 µm, and (**g**,**h**) >20 µm against experiment day for ND (**left**) and NR (**right**). The individual data points (filled circles) from each bag are overlain by the GAMM fits (mean and 95% Confidence Interval) for the control (green) and treatments (*pH*, orange and *pH*/*T*, red), with the three phases of the experiments delineated by the vertical lines. Note that the Chl-*a* concentration scale on the *y*-axis differs between experiments.

**Figure 7:**
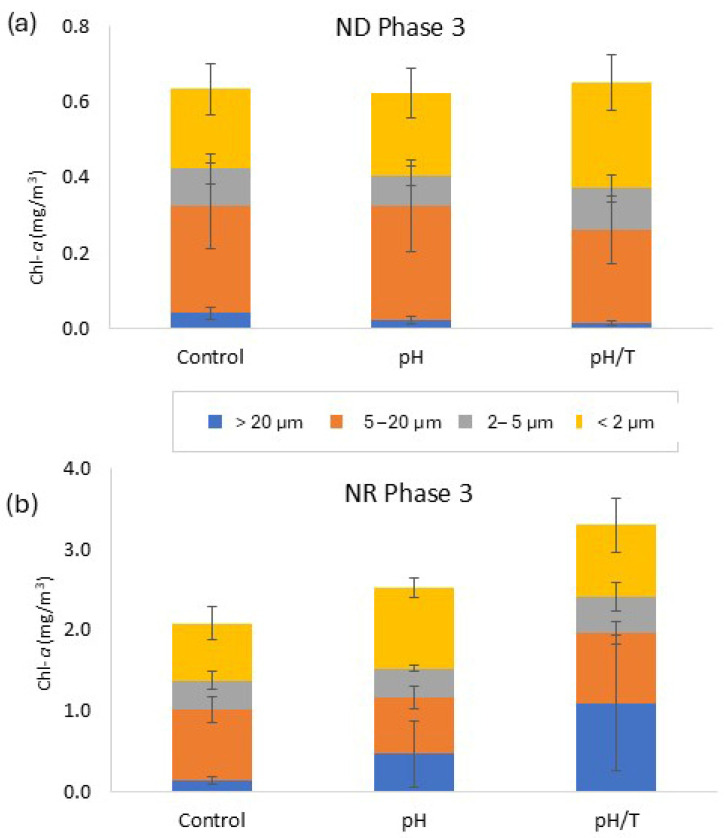
Chl-*a* size fractions (mg/m^3^, mean ± 1 standard deviation) in Phase 3 for (**a**) ND and (**b**) NR. Note that the Chl-*a* concentration scale on the *y*-axis differs between experiments.

Measurement of different Chl-*a* size fractions provided insight into phytoplankton size spectrum response to the treatments (Figure 6 and Figure 7). In ND the 2–5 µm fraction was lower in *pH* relative to the control (*p <* 0.01) during Phase 2 (Figure 6c), with evidence of a change in phenology (Table S1), whereas the 5–20 µm fraction exhibited a higher maximum in *pH*/*T* during Phase 2 (Figure 6e) that corresponded to the total Chl-*a* maximum (Figure 5a). Conversely, the >20 µm fraction in Phase 3 in both treatments was lower than in the control (Figure 6g,h). In NR both treatments induced a change in phenology for the 0.2–2 µm fraction (*p* < 0.001; Table S1; Figure 6b). The 5–20 µm fraction was lower in *pH* relative to the control in Phase 3 (*p* < 0.01) with a different temporal trend (*p* = 0.006; Table S1, Figure 6f). In Phase 3 of NR there was a larger > 20 µm fraction in both treatments (*pH* 0.47 mg/m^3^; *pH*/*T* 1.1 mg/m^3^) relative to the control (0.14 mg/m^3^, Figure 6h), in contrast to the lower > 20 µm fraction in both treatments relative to the control in ND (Figure 7).

#### 3.2.4. Phytoplankton Community Composition

**Figure 8:**
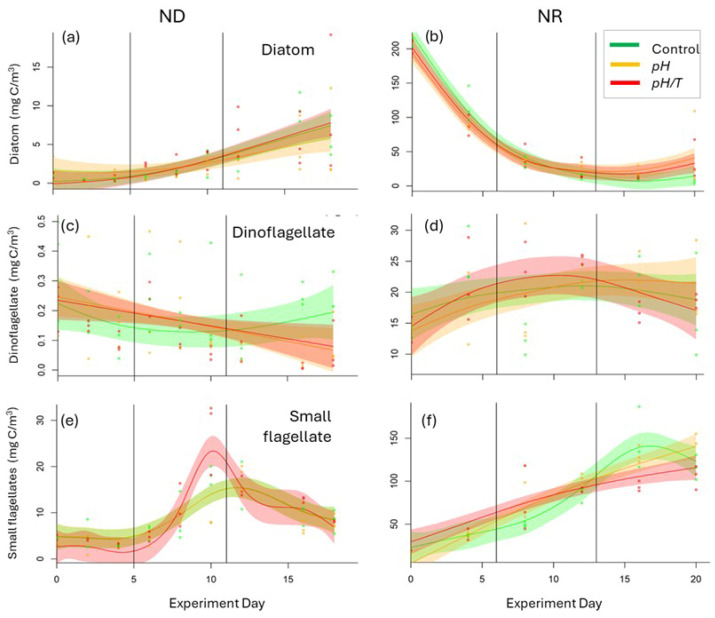
Biomass (mg C/m^3^) of (**a**,**b**) diatoms, (**c**,**d**) dinoflagellates, and (**e**,**f**) small flagellates against experiment day for ND (**left**) and NR (**right**). The individual data points (filled circles) from each bag are overlain by the GAMM fits (mean and 95% Confidence Interval) for the control (green) and treatments (*pH*, orange and *pH*/*T*, red), with the three experiment phases delineated by the vertical lines. Note that the Chl-*a* concentration scale on the *y*-axis differs between experiments.

**Figure 9:**
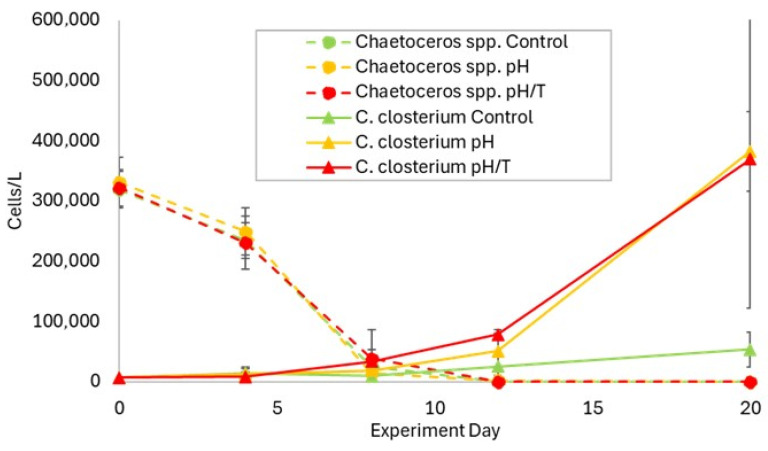
Cell abundance of two dominant diatom species, *Chaetoceros* spp. and *Cylindrotheca closterium* in the control (green) and treatments (*pH*, orange and *pH/T*, red) against experiment day during NR.

Although diatoms represented a major component of the initial phytoplankton biomass in ND and increased to 19 mg C/m^3^ by the end of the experiment, their biomass showed no treatment effect under nutrient-deplete conditions in ND (Figure 8a). The pennate diatom *Cylindrotheca closterium* dominated the diatom community, with *Thalassionema nitzschioides* and *Thalassiosira minima* contributing most of the residual diatom biomass. Conversely, NR started during a diatom bloom (>200 mg C/m^3^), with biomass declining sharply over the first 7 days to <20% of the initial value (Figure 8b) and corresponding decreases in eight of the dominant diatom species, including *Chaetoceros* spp., and *Lauderia* spp. However, the diatoms exhibited phenologic differences in both treatments in NR (Table S1), with higher biomass in Phase 3 (*pH* 33.5 mg C/m^3^, *p* = 0.03; *pH*/*T* 24.7 mg C/m^3^, control 8.36 mg/m^3^; *p* = 0.01) as a result of the increase in *C. closterium* (Figure 9).

In ND the dinoflagellates accounted for a minor proportion of phytoplankton biomass and declined throughout (Figure 8c). However, despite variability between replicates there was lower dinoflagellate biomass in both treatments (*pH* 0.07 mg C/m^3^, *p* = 0.02; *pH/T* 0.06 mg C/m^3^, *p* = 0.003) in Phase 3 relative to the control (0.22 mg C/m^3^) in ND. Dinoflagellate biomass was initially higher in NR at 12–15 mg C/m^3^ and increased to 20–30 mg C/m^3^ by the end of the experiment (Figure 8d), with *Gymnodinium* spp. and *Prorocentrum minimum* dominating. However, variability between replicates precluded determination of a treatment effect on dinoflagellates in NR.

Small flagellates initially dominated phytoplankton biomass in ND and increased from 5 mg C/m^3^ to a maximum of >30 mg C/m^3^ in *pH*/*T* during Phase 2 (Figure 8e), with a treatment effect on phenology (*p* < 0.001, Table S1). This increase in the small flagellates in *pH*/*T* corresponded to the response of Chl-*a* (Figure 5a) and also the 5–20 µm fraction (Figure 6e). Although initially a minor component of the phytoplankton in NR, small flagellate biomass increased monotonically throughout the experiment (Figure 8f). However, the treatment response in NR was the converse of ND, with a lower Phase 3 biomass (*pH*/*T* 99.6 ± 11.3 mg C/m^3^, control 135.8 ± 37.1 mg C/m^3^, *p* = 0.08). The biomass of other phytoplankton groups, including the raphidophytes, silicoflagellates and other flagellates was higher in NR relative to ND, but none of these groups exhibited treatment effects.

#### 3.2.5. Particulate Elemental Composition

Although Particulate Carbon (PC) decreased throughout ND, there were no differences in PC, PN or Carbon:Nitrogen (C:N) between the treatments and control. PC, PN, and C:N also decreased during NR, from initial high values associated with the bloom, but again there were no treatment effects (Table 2 and Figure S2).

## 4. Discussion

### 4.1. Chl-a Response to Lower pH

A wide variety of phytoplankton responses to ocean acidification have been reported in both magnitude and sign [44,45], limiting predictive capacity and highlighting the need to establish regional responses. Contrary to other observations [24,25], this study showed no change in Chl-*a* under lower pH alone (Figure 4; Table 2), which is surprising given that the corresponding increase in pCO_2_ might be expected to alleviate the metabolic costs of maintaining carbon supply to the cell [5]. However, other coastal studies have also reported minor or no chlorophyll-*a* response to lower pH [46,47], which may reflect high physiological plasticity or intraspecific diversity [48]. Exposure to environmental variability has been suggested to promote adaptation in phytoplankton [43], with negligible response to lower pH often attributed to in situ exposure to low and variable pH in coastal waters [2,46,49]. The parallel time series measurements and mesocosm experiments in this study enabled assessment of this hypothesis. pH time series are rarely reported with ocean acidification experiments, despite the fact that “*comprehensive accounting of both mean and extreme carbonate system conditions…is necessary to understand how coastal marine communities will respond to future acidification challenges*” [23]. Documenting inter- and intra-annual variability not only establishes limits for experimental treatments and avoids extreme pH perturbations [50] but also increases relevance when combined with regional climate projections [30]. The pH trend during the four-year time series (Figure 2) is similar to that of other New Zealand coastal sites but with generally lower inter-annual variability compared to national and international sites [19,31,51]. This suggests that, despite elevated residence time in the semi-enclosed Ākautangi Evans Bay, the pH is primarily modulated by oceanic water input from Cook Strait (Figure 1a). Critically, the pH minima of 7.85 during the 4-year time series was higher than the respective treatment pH of 7.65 and 7.75 in the two experiments (Table 1, Figure 2b), confirming that predicted acidification by the end of the century will exceed the current pH range in these waters. Furthermore, the ambient pH always exceeded 8.0 during the seasons when the two experiments were carried out. In addition, diurnal pH variability in the bay water was relatively low during both experiments compared to other global time series sites [19]. Consequently, both the long-term time series pH record and within-experiment pH variation in the ambient water indicate that pre-exposure of the phytoplankton community to lower pH is not the reason for the observed lack of response of Chl-*a* in the *pH* treatments.

### 4.2. Chl-a Response to Lower pH and Warming

The two experiments exhibited differential responses to the two treatments in specific parameters (Table 3). Neither experiment was full factorial as there was no warming-only treatment, in part because this scenario is not relevant to the future where warming will be inextricably linked with lower pH [14]. However, the absence of a warming-only treatment precluded determination of the mode of interaction between the two climate drivers [16,52]. In contrast to the lower pH treatment, combined warming and lower pH resulted in higher Chl-*a*, as previously reported in other studies [16,53,54]. This response is also consistent with warming-only studies that show enhanced Chl-*a* at elevated temperatures [55]. An increase in ambient temperature may accelerate metabolic rates and could account for the earlier and larger Chl-*a* maximum during Phase 2 in the combined treatment in ND (Figure 5a), particularly if accompanied by an increase in heterotrophic nutrient regeneration as reported under lower pH and warming [56]. Alternatively, the Chl-*a* response in the combined treatment may reflect more favourable temperature and/or greater thermal tolerance in relation to the temperature reaction norm of the dominant phytoplankton species [10]. Conversely, decreases in phytoplankton biomass at warmer temperatures [57,58], have been attributed to top–down control via enhanced zooplankton grazing [59]. The large-volume mesocosms facilitated prey–grazer interaction and species competition, and so grazing may have been a factor in the phytoplankton response; however, assessment of grazing was limited to determination of zooplankton diversity and diet, based upon fatty acid profiles, in NR, both of which showed no treatment effect [28]. Overall, the Chl-*a* maximum was higher under combined low pH and higher temperature in both experiments, as reported in other studies [60], indicating that phytoplankton growth rate exceeded grazing. Although the reason for Chl-*a* accumulation cannot be confirmed, the differing response between the treatments in both experiments highlights the importance of altering warming and pH in combination for prediction of the future status of coastal phytoplankton.

### 4.3. Cell Size Response

Temperature is a key determinant of cell size [13,61], but this may be modulated by CO_2_ availability. Although certain phytoplankton size fractions showed a significant response to lower pH alone, overall, there were more responses to the combined treatment in both experiments (Table 3). An increase in smaller phytoplankton under lower pH has often been reported [25,62,63], and this may be enhanced under higher temperature [16,55]. In addition, elevated metabolic activity at warmer temperatures may have accelerated nutrient-limiting conditions in ND, benefitting smaller cells with their advantageous cell morphometry and lower nutrient requirements, which may explain the increase in the 0.2–2 µm size fraction in the combined lower pH and warming treatment in ND (Figure 6a). Conversely, in NR the >20 µm fraction increased in both treatments (Figure 6h), inferring that lower pH was the primary determinant of this response when nutrients were available. As larger cells are more diffusion-limited with respect to nutrient and carbon supply, this suggests that alleviation of carbon limitation under lower pH [11,64] may occur more readily when nutrients are available, as in NR (Figure 6h, Table 2), whereas larger cells may be outcompeted by smaller cells under nutrient-limited conditions [62], as in ND (Figure 6g). Although increases in phytoplankton biomass have been reported under lower pH and low nutrient concentrations [24,63], nutrient availability is generally considered to limit phytoplankton response to ocean acidification [54,65]. The results of this study also suggest that nutrient availability may modulate cell size response to combined warming and lower pH; however, this interpretation is limited by the differing initial community composition and bloom status of the two experiments which may have influenced response regardless of nutrient status. A further caveat is the different initial temperature of the two experiments, with warming in *pH*/*T* potentially resulting in a more optimal temperature for the dominant phytoplankton species in NR. Acknowledging these limitations, the current study nevertheless supports the increasing recognition of nutrient availability as a determinant of phytoplankton response to future conditions [3,4,17,66].

### 4.4. Diatom Response

The diatoms have been a primary focus in ocean acidification and warming experimental studies, as this ubiquitous phytoplankton group accounts for half of marine primary production and plays a major role in the marine biogeochemical cycles and carbon export [45,67]. As diatoms generally utilise CCMs, they frequently respond positively to the alleviation of carbon limitation under lower pH [68], resulting in diatom domination of the phytoplankton community and associated changes in cell size spectrum. Diatoms are also generally favoured by elevated temperatures, with warming experiments showing corresponding increases in larger size fractions and faster bloom development [14,66]. These observations concur with the observed increase in diatoms and larger cells during Phase 3 in both *pH* and *pH*/*T* treatments in NR (Figure 8b), which suggests that increased carbon availability is the primary driver of the diatom response. Conversely, the diatoms did not respond in either treatment in ND (Figure 8a; Table 1), which may be interpreted as nutrient availability modulating response to lower pH [18,69], although the responses in ND and NR cannot be directly compared due to differing initial conditions, as previously discussed. Although some studies suggest increased CO_2_ availability may alleviate nitrogen limitation in diatoms [70], diatom photosynthesis and respiration decline under lower pH in N-deplete cultures, in contrast to N-replete cultures [69]. Li et al. [17] suggest that although lower pH may increase carbon availability, when combined with nutrient limitation this can impact cellular transport mechanisms and dissolved inorganic supply to the cell. Consequently, although larger diatoms may benefit from future acidification [45,71], this response may be restricted to nutrient-replete conditions, which could result in asymmetry in diatom abundance and biomass between the oceans, where warming-driven stratification may reduce nutrient availability, and coastal waters where eutrophication is increasing [72].

During Phase 2 of ND the Chl-*a* maximum (Figure 5a) coincided with an increase in nitrate and ammonium concentration in the control and treatments (Figure 4a,c), indicative of heterotrophic regeneration [24]. The initial silicic acid concentrations of ~1.5 µmol/L remained stable until the mid-point of ND (Figure 4g) with the increase in dissolved inorganic nitrogen supporting silicic acid uptake. Conversely, silicic acid concentrations in NR were initially low (Figure 4h) due to the bloom at the start of the experiment, which would have limited diatom growth. Although the water in the experiments was isolated from natural replenishment of nutrients from sediments, freshwater and mixing, the nutrient additions throughout NR elevated concentrations to match ambient levels (Table 1; Figure 4b,d,f,h). As a result, silicic acid concentrations increased to 1.7–3.0 µmol/L by the end of NR and supported an increase in diatom biomass in both treatments in Phase 3. This is a similar response to nutrient addition under lower pH in other mesocosm experiments [24,26], but unlike those studies the maintenance of ambient nutrient concentrations in NR simulated the natural recovery of the phytoplankton community following bloom-driven nutrient depletion.

The diatoms are characterised by a range of sizes, morphologies and ecological niches [45,67,73], and so it is perhaps unrealistic to expect this highly diverse group to display a uniform response to lower pH. However, this diversity engenders broad physiological plasticity that enables niche exploitation, and so future conditions may be more optimal for certain diatom species. This is exemplified by the positive responses of *Skeletonema* and *Chaetoceros* sp. to lower pH, with or without warming [14,71]. However, nutrient availability may also play a role, with the large centric *Coscinodiscus* spp. benefiting under nutrient-deplete lower-pH conditions [62], and the pennate *Guinardia striata* responding to lower pH when nutrients were available [3]. In this study, the pennate diatom *C. closterium* was initially present in ND but did not respond in either treatment under nutrient-deplete conditions, whereas it increased abundance in both treatments in Phase 3 of the nutrient-replete NR (Figure 9). Although *C. closterium* has a cell diameter of ~5 µm, its cell length of 30–300 µm, combined with the tendency for cells to clump as a result of mucilage production, may account for the corresponding increase in the >20 µm fraction in NR (Figure 6h). This dominance of a pennate diatom species is consistent with some ocean acidification studies [71], but contrasts with findings of others in which pennates declined relative to centric diatoms under lower pH [74], and combined lower pH and warming [16]. *C. closterium* is a ubiquitous diatom species with a high growth rate [75] and exhibits flexibility in trophic mode [76]. This species may then have a competitive advantage under conditions of greater nutrient variability in coastal waters, in contrast to the more stable nutrient concentrations in the oceans where centric diatoms may be more competitive and respond to lower pH [16,74]. Consequently, following the depletion of nutrients and particularly silicic acid (Figure 4g), during the bloom at the start of NR, *C. closterium* outcompeted other diatom species when nutrient availability increased, consistent with observations of post-bloom domination by fast-growing species in other ocean acidification experiments [24]. As centric and pennate diatoms have differential effects on trophic interactions and carbon sequestration [67], this highlights the importance of maintaining nutrient availability at ambient concentrations for realistic projection of regional response to future change.

### 4.5. Dinoflagellate and Flagellate Response

The low dinoflagellate biomass in ND and variability between replicates in NR limits conclusions regarding the response of this group to future conditions. Dinoflagellates might be considered to have an advantage in the nutrient-deplete conditions in ND, yet their biomass decreased in the treatments. This could reflect the elevated Si:N in ND which may have favoured the diatoms [77] and resulted in the decline in the dinoflagellates from the mid-point of the experiment (Figure 8c). Conversely, the small flagellates increased in biomass throughout both experiments (Figure 8e,f). Although their initial biomass was lower in ND, their smaller size enabled more effective competition for the limited nutrients, which may account for the larger and earlier maximum in the small flagellates in the combined lower pH and warming treatment during Phase 2 that coincided with the Chl-*a* maximum (Figure 5). The subsequent decline in the small flagellates from the mid-point of ND onwards, and also in Phase 3 of NR (Figure 8f), suggests that, as with the dinoflagellates, the small flagellates may have been outcompeted by the diatoms. If so, then this competition was influenced by nutrient availability, further emphasising the importance of using regionally relevant nutrient concentrations in future perturbation experiments. As small flagellates are an important sink for bacteria, future decreases in their biomass may influence bacterial abundance and processes in coastal waters. Furthermore, the relationship between small flagellates and dimethylsulphoniopropionate concentrations reported in these experiments [27] suggests that future declines in small flagellates may alter coastal emissions of dimethylsulphide (DMS) and potentially influence aerosol formation.

### 4.6. Implications for Coastal Ecosystems

Although phytoplankton blooms may occur earlier under warmer temperatures [53,57,58,78], the observed two-day shift in Chl-*a* in ND (Figure 5a) is relatively minor and within the inter-annual variability of surface Chl-*a* in the Wellington region [79]. As a greater proportion of larger phytoplankton reduces food web transfers and increases trophic transfer efficiency [80], the increase in larger pennate diatoms under predicted future conditions suggests potential for increased carbon and energy flow in temperate coastal food webs. However, in contrast to reported increases in particulate C:N under lower pH [69,81,82], there was no treatment effect on particulate C:N in NR. Furthermore, whereas consumers might benefit from an increase in large cells due to diet size specificity, there were no treatment effects observed in copepod fatty acid content or in zooplankton community composition during NR [28]. This may reflect that the experiment duration of 18–20 days was short relative to the generation time of approximately one month for copepods. Regardless, whereas larger diatom species and Chl-*a* concentration increased under future nutrient-replete conditions, changes to carbon and energy flow in temperate New Zealand coastal food webs may be moderate.

## 5. Conclusions

Overall, the results indicate that the base of the food web in temperate New Zealand coastal waters may be resilient to projected future changes in pH and temperature. Combined warming and lower pH resulted in an increase in total Chl-*a*, with lower pH the primary driver of increases in diatom biomass and the >20 µm size fraction when nutrients were available. In addition to demonstrating that predicted pH changes for the end of the century exceed current extremes in coastal waters, the parallel in situ pH time series also provided evidence that the absence of a Chl-*a* response to lower pH did not result from previous in situ exposure. The results also suggest that experiments on natural communities that focus solely on ocean acidification may be of limited value as the coincident warming, and its interaction with lower pH [15,18], can have different effects. This highlights that the integrated impact of multiple climate drivers [14] on natural communities should be determined for a more robust basis for prediction of future coastal phytoplankton ecosystems.

The results indicate that future changes in pH and temperature may not alter phytoplankton nutrient uptake in coastal waters. Although the influence of other factors, such as zooplankton grazing, viral lysis and bacterial processes, were not characterised in this study and may have influenced response, the inferred role of nutrient availability in modulating phytoplankton response to future conditions has implications for experimental assessment and prediction of regional climate change impacts. Although it is established that phytoplankton are sensitive to nutrient availability, warming and acidification, there have been few experimental studies of the interaction of these stressors [20,66], and specifically in which ambient nutrient concentrations are maintained. Consequently, further multi-stressor experiments using regionally relevant nutrient availability are recommended for both coastal water management [21] and prediction of future change.

## Figures and Tables

**Table 1:** Details of the mesocosm experiments ND and NR, including initial conditions, treatments and duration, and initial nutrient concentrations and additions. NO_3_: Nitrate–nitrogen, NH_4_: ammonium–nitrogen, P: phosphate, Si: silicic acid.

Exp.	Time	Initial Temp (°C)	Initial pH	Initial Chl-*a* mg/m^3^	Treatment (*pH, pH/T*)	Days	Mean Initial Nutrients (µmol/L) NO_3_/NH_4_/P/Si	Nutrient Addition (µmol/L) During exp. NO_3_/NH_4_/P/Si
ND	Autumn 2016	17.1	8.03	0.8	−0.33 −0.33/+2.5 °C	18	0.4/0.8/0.4/2.5	Nutrient-deplete No addition
NR	Spring 2018	12.1	8.15	7.0	−0.5 −0.5/+3.5 °C	20	0.3/0.3/0.2/0.6	Nutrient-replete Daily addition Total: 1.8/1.8/0.9/2.0

**Table 2:** Particulate carbon and nitrogen concentrations, and C:N ratios (mean and standard deviation, sample number = 3) in Phase 3.

Particulate		ND	NR
Carbon (mmol/m^3^)	Control	11.91 ± 2.2	23.1 ± 2.3
	*pH*	12.28 ± 2.6	21.83 ± 2.3
	*pH*/*T*	13.28 ± 2.9	27.25 ± 3.9
Nitrogen (mmol/m^3^)	Control	1.16 ± 0.35	4.21 ± 0.2
	*pH*	1.14 ± 0.35	4.17 ± 0.2
	*pH*/*T*	1.31 ± 0.41	3.83 ± 0.4
C:N (mol)	Control	10.2 ± 2.2	5.5 ± 0.3
	*pH*	10.7 ± 3.1	5.2 ± 0.4
	*pH*/*T*	10.1 ± 1.8	7.1 ± 0.7

**Table 3:** Summary of treatment effects (*pH*, orange; *pH*/*T*, red) relative to the controls in ND and NR. Treatment effects on phenology, as determined by GAMMS (*p* < 0.01, Table S1), are indicated by a star and differences in Phase 3 mean value relative to the control by arrows with the direction indicating sign of treatment response.

	Experiment	ND Nutrient-Deplete		NR Nutrient-Replete	
	Treatment	*pH*	*pH/T*	*pH*	*pH/T*
Chl-*a* Size fraction	Total		* 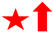 *		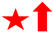
	0.2–2 µm		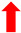	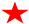	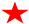
	2–5 µm	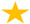			
	5–20 µm		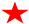		
	>20 µm	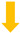	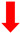	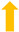	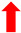
Group	Diatoms			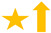	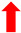
	Dinoflagellates	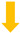	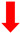		
	Small flagellates		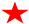		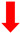

## Data Availability

The original contributions presented in this study are included in the article/supplementary material. Further inquiries can be directed to the corresponding author.

## Supplementary Materials

The following supporting information can be downloaded at https://www.mdpi.com/article/10.3390/microorganisms14050989/s1. Figure S1. Temperature and pH in Evans Bay during the 4-year time series (2015–2019); Table S1. Summary of GAMMS analysis; Figure S2. Response of Particulate Carbon and Nitrogen concentrations.
