# Supplementary material for: Coastal Phytoplankton Response to Acidification and Warming Under Differing Levels of Nutrient Availability
Source: Microorganisms. 2026 Apr 28;14(5):989. doi: 10.3390/microorganisms14050989 (PMC13209521; doi:10.3390/microorganisms14050989)
Supplement: Supplementary file 1 [file microorganisms-14-00989-s001.zip › microorganisms-4222813-supplementary.pdf]

Supplementary Information

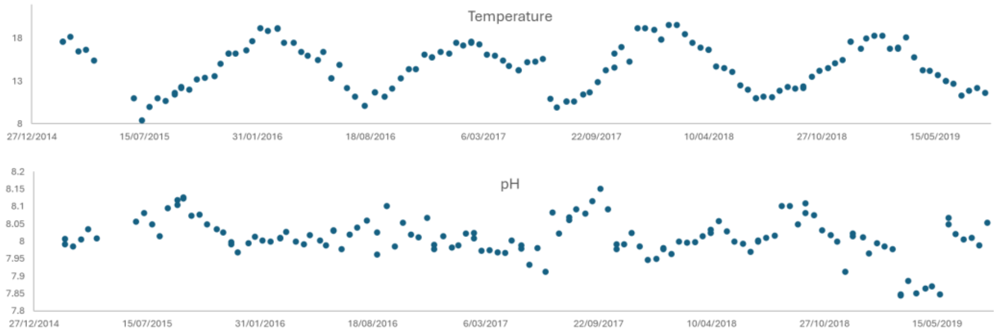

Figure S1. Temperature and pH collected at the water intake in Evans Bay at bimonthly intervals during the 4-year time series (2015-2019) that encompassed the mesocosm experiments.

Table S1. Summary of GAMMS analysis

**ND**					p-value	p-value
Parameter	Model 1	Model 2	Model 3	Lowest AIC	*pH*	*pH/T*
Total Chl-a	-19.494	-25.977	-29.350	Model 3	0.344	< 0.001
0.2-2 µm	-187.598	-155.753	-185.069	Model 3	0.472	0.254
2-5 µm	-348.316	-349.718	-356.901	Model 3	0.009	0.133
5-20 µm	-42.494	-54.683	-66.675	Model 3	0.748	< 0.001
>20 µm	-484.413	-470.773	-484.352	Model 1 & 3	0.675	0.060
Diatoms	384.429	387.825	384.836	Model 1 & 3	0.152	0.752
Dinoflagellates	-117.134	-118.246	-118.246	Model 2 & 3	0.074	0.126
Small flagellates	459.316	447.253	445.306	Model 3	0.889	< 0.001

**NR**					p-value	p-value
Parameter	Model 1	Model 2	Model 3	Lowest AIC	*pH*	*pH/T*
Total Chl-a	170.758	156.850	148.514	Model 3	0.108	< 0.001
0.2-2 µm	-39.634	-44.711	-43.929	Model 2 & 3	< 0.001	< 0.001
2-5 µm	-128.263	-131.214	-135.730	Model 3	0.398	0.036
5-20 µm	-88.883	-98.654	-98.331	Model 2 & 3	0.006	0.980
>20 µm	106.137	111.808	106.119	Model 1 & 3	0.544	0.070
Diatoms	476.864	476.010	470.069	Model 3	< 0.001	0.033
Dinoflagellates	336.350	340.778	338.922	Model 1	0.298	0.525
Small flagellates	485.618	474.898	473.620	Model 2 & 3	0.374	0.063

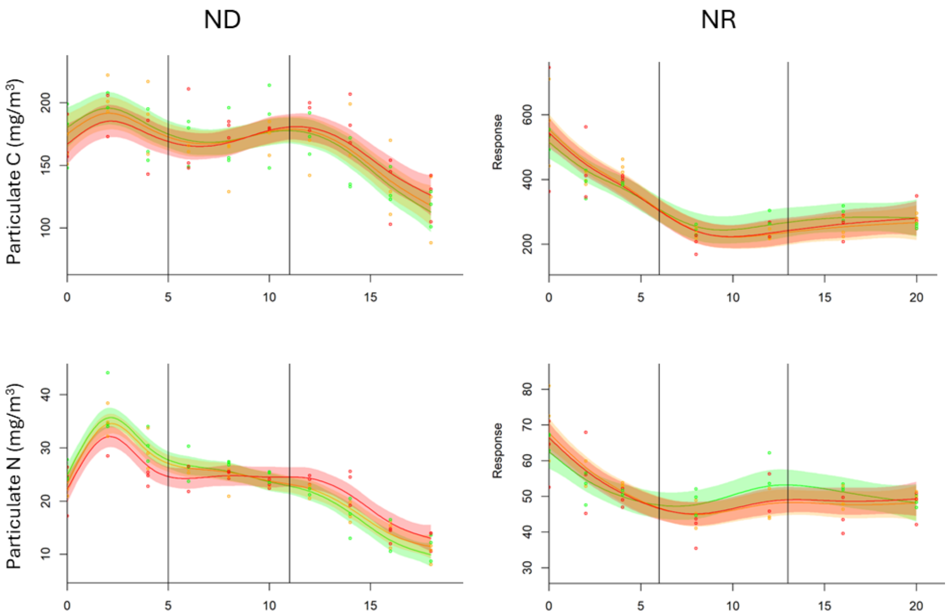

Figure S2. Response of Particulate Carbon and Nitrogen concentrations (mg/m^3) in ND (left) and NR (right), with the individual data points from each replicate overlain by the GAMM fits (mean and 95% Confidence Interval) for the Control (green) and treatments (*pH*, orange and *pH/T*, pink), with the different experiment phases delineated by the vertical lines.
